# Longitudinal event-sampling dataset of football fans across five countries during the 2024 European championship

**DOI:** 10.1038/s41597-026-07643-z

**Published:** 2026-06-23

**Authors:** Hannes M. Petrowsky, Yannik A. Escher, Lea Boecker, Kathi Diel, Paweł Muniak, Meikel Neumann, Danna Oomen, Leonie Schwarz, Annik Strauch, Marcel Weber, Malte Friese, Oliver Genschow, David D. Loschelder

**Affiliations:** 1 Institute of Management and Psychology, Leuphana University, Lüneburg, Germany; 2 Department of Psychology, Saarland University, Saarbrücken, Germany; 3 Centre for Research on Social Relations, SWPS University of Social Sciences and Humanities, Warsaw, Poland

## Abstract

This dataset was collected during the UEFA EURO football championship in 2024. It provides match-contingent experience sampling data, including (a) individual-level variables such as life satisfaction, (b) interpersonal variables such as prosocial intent, and (c) broader societal variables such as political trust. The data points are nested in a sample of *N* = 1,012 individuals from five countries (England, Germany, Italy, the Netherlands, Poland). Using an event-sampling method with four to eight measurement waves (depending on how far the national team progressed in the tournament), we captured time-sensitive fluctuations in a total of *m* = 30 variables in response to the national team’s objective (i.e., win, draw, loss) and perceived success. In addition, the dataset contains moderating variables including Big-5 personality traits, conspiracy and free-will beliefs, or alcohol consumption and betting behaviors, and detailed match-specific data (e.g., shots on target, yellow cards, attendance). Our data descriptor portrays the methodological approach, data collection procedures, and available variables, and outlines how the data may be used for future research.

## Background & Summary

The present longitudinal, event-sampling data was collected during the multinational UEFA EURO championship in the summer of 2024. Multinational sport events are widely recognized as having positive as well as negative psychological, societal, political, and financial impacts for host countries^1^: The manifold benefits range from increased quality of life^2^ to boosted tourism and economic growth^3^, and from improved national well-being^4,5^ to more sports participation^6^. On the other hand, these sports events also involve high economic and social burdens for residents and taxpayers^7,8^ and pose substantial environmental risks^9^. While much of the available research focused on the long-term effects of hosting tournaments^10–12^, surprisingly little data is available on the immediate, potentially profound impact of international sports events on individual spectators’ feelings, thoughts, and their socio-political attitudes.

To examine these immediate effects of an omnipresent international sports event on both proximal and distal psychological, social, and societal outcomes, we carried out a large-scale experience sampling study during the course of the men’s European Championship of Football (i.e., the UEFA EURO 2024). This tournament attracted 8.9 million spectators in the stadiums and fan zones, and over 5 billion television viewers around the world^13^. Using a preregistered event sampling method with an extensive baseline assessment and three to seven subsequent measurement waves (dependent on national team’s tournament progress), we recruited spectators from five different countries (*n* ≥ 200 per country, i.e., total *N* = 1,012) and captured their time-sensitive responses to their national teams’ match outcomes.

**Fig. 1:**
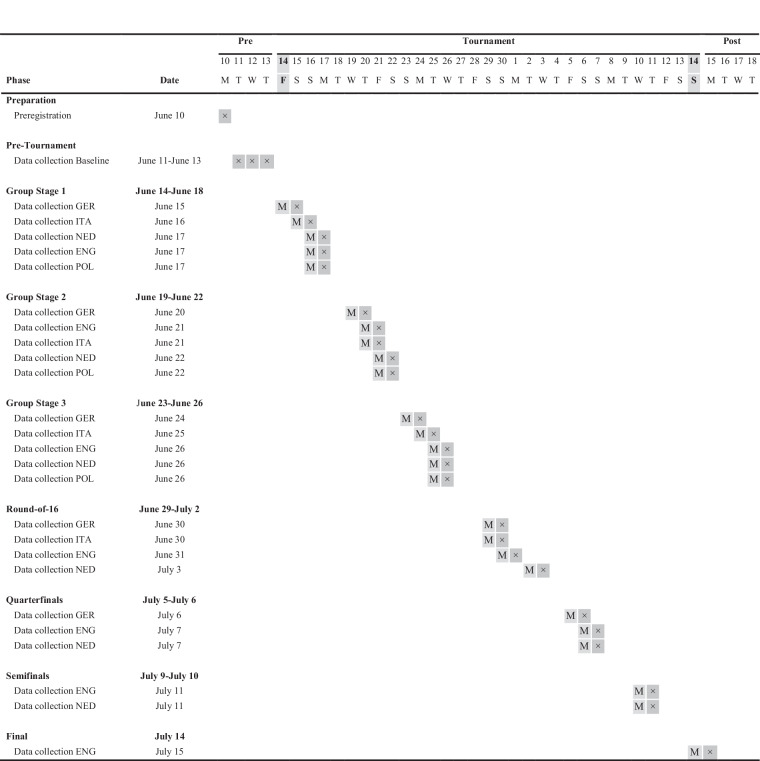
Data Collection Procedure Across the Four-Week Data Collection Period. The data collection of the present data set spanned four weeks during the UEFA EURO 2024 that took place in Germany from June 14th to July 14th. Match days are displayed as ‘M’ with light gray background. Data collection days are displayed as ‘×’ with dark gray background. In total, we followed 25 matches from five national teams. ENG = England, GER = Germany, ITA = Italy, NED = Netherlands, POL = Poland.

Our event-sampling data follows a multi-level structure with three levels: (1) countries, (2) individuals, and (3) events (i.e., matches). On the (1) country level, we collected data from spectators residing in Germany, England, the Netherlands, Italy, and Poland via Prolific. All countries participated in the UEFA EURO 2024 that took place in Germany from June 14th to July 14th (see data collection procedure in Fig. 1). The country selection was based on the number of eligible participants on Prolific and on past UEFA EURO championship victories (i.e., Germany: three, Italy: two, the Netherlands: one, England and Poland: none), aiming to maximize the chance of generating data points for all possible match outcomes (i.e., wins, draws, and losses) throughout the tournament. More specifically, we expected the different countries to drop out at different stages of the tournament, leading to variations in match outcomes and ideally a (roughly) equal distribution of wins vs. losses in the dataset. In all, our final dataset is slightly imbalanced but covers a total of 11 wins, 6 draws, and 8 losses across all countries. As expected, draws were least prevalent given that the tournament format inevitably precludes any draws upon completion of the initial group stage.

On the (2) individual level, we collected longitudinal event-contingent data from the spectators of the UEFA EURO 2024. The data collection involved multiple measurement waves and individual participants completed both (a) an extensive survey of background variables in the baseline questionnaire one week prior to the tournament and (b) up to seven event-specific measurement waves the day after each match of their national team.

Finally, on the (3) event level, we objectively scored match-related information and outcomes. Based on publicly available data, we included, for example, objective performance measures, difference scores between the participating countries (e.g., world ranking differences), or betting odds (detailed below). In sum, the dataset encompasses country-level, individual-level, and event-level data across the UEFA EURO tournament period of four weeks. The preregistered design for the data collection and the primary dataset^14^ are openly available on the Open Science Framework (OSF) repository (10.17605/osf.io/f84g3).

The present dataset has not yet been used in any prior publications. Our data examine the (ir-)relevance of sports events and match outcomes for shaping societal attitudes, psychological perceptions, as well as the development of social perceptions and behaviors. It can be utilized to uncover potential cultural, cross-country differences in affects, behaviors, and cognitions in spectators’ responses to collective national sports success. As such, the present data will be of interest for researchers of various disciplines in the human and social sciences, including psychology, political science, or sociology, among others. The present data will also be of interest for scholars in sports research, including sports management, sports marketing, and sports consumption. The data can provide empirical evidence for different predictions from different theoretical lenses and includes a variety of variables pertaining to emotions, social identity, group processes, cultural differences, political attitudes, or sexual behavior. The data furthermore offer ample opportunities for researchers interested in advanced statistical methodologies, such as time-series analyses or multilevel modeling.

## Methods

### Transparency and ethical approval

All data^14^ and supplementary material are publicly available on the OSF. We preregistered the full design and procedure prior to data collection (https://osf.io/a3mrq). Participants’ informed consent was obtained prior to the study. Specifically, participants confirmed having understood that (a) their participation is completely voluntary, (b) they can withdraw at any point without expecting disadvantages, (c) data are anonymous, and (d) the anonymized data will be made publicly available. Participants gave informed consent prior to the baseline questionnaire, as well as prior to each and every event questionnaire.

We adhered to the ethical guidelines of our research institution for good scientific practice, and the study was in line with the Declaration of Helsinki. The study’s experimental protocols including the use of human participants were conducted in accordance with the ethical guidelines of the Research Commission of the School of Management and Technology at Leuphana University Lüneburg, which issued a formal exemption from ethical review. The exemption was granted because the study met all required criteria for non-invasive and non-stressful survey research, in line with the ethical standards of the German Research Foundation (DFG), the American Psychological Association (APA), and other relevant guidelines for research including human participants. We matched the participants’ data using their completely anonymous, self-reported Prolific ID (i.e., an alpha-numerical code with 24 random digits). For the publicly available datasets, we removed this identifier from the data and replaced it with a sequential number. By doing this, the data never allowed for the identification of individual participants or the disclosure of sensitive data. The data were stored locally and only one member from the author team had access to the data including the Prolific identifier.

### Design and sample

We collected data from a total of *N = *1,012 spectators from five different countries before and during the UEFA EURO tournament. Prior to the tournament, participants completed a baseline questionnaire that captured general information (e.g., demographics) and trait-level moderators (detailed below). This initial survey was conducted in the week before the tournament started (June 10 through June 14, 2024). Participants were transparently informed about the overarching goal of this data collection, about the event sampling procedure for the following weeks, and about their remuneration.

Participants provided the rest of these longitudinal data during the subsequent four weeks of the tournament in an event-contingent experience sampling design^15^. Specifically, we asked participants to fill out questionnaires after each match of their respective national team—that is, “whenever events meeting a predetermined definition have occurred” (p. 199). The predetermined definition for the present research was that participants’ football team from their home country had played a tournament match on the previous day. The event sampling method hence allowed to investigate the persistence, change, and temporal structure of event-related thoughts, affects, and behaviors^16^.

We collected event data in three to seven measurement waves to capture time-sensitive fluctuations in response to the tournament matches (please refer to Table 1 for participation rates per measurement wave and country and Table 2 for completion rates on participant-level). More concretely, participants were contacted the day after each match their national team played at 12:00 p.m. local time. We standardized the questionnaire time to 12:00 p.m. on the following day to ensure that effects were not driven by differences in kick-off times (e.g., 3:00 p.m. vs. 9:00 p.m.). All information messages and questionnaire items were formulated in the national language of the respective country and proofread by a native speaker. Event-related surveys included *m* = 30 variables assessing affective, behavioral, and cognitive aspects. The order of variables was randomized between events and participants to prevent any carryover effects. In addition to these self-report measures, we objectively coded event-related outcomes and background information (e.g., shots on target, fouls, attendance).

**Table 1:** Participation Rate (in %) and Match Results Across Countries.

Event	England (*n* = 202)	Netherlands (*n* = 204)	Germany (*n* = 204)	Italy (*n* = 201)	Poland (*n* = 201)
Preliminary round					
Match 1	88.6	77.0	63.2_a_	73.6_b_	84.1
Match 2	87.6	54.4_a_	70.1	76.1	63.7_a_
Match 3	80.7	63.2	67.2	81.1	75.1
Knock-out round					
Round-of-16	82.2	63.7	67.2_b_	72.1_b_	—
Quarterfinals	65.8_b_	52.9_b_	60.8_a_	—	—
Semifinals	80.7	57.8	—	—	—
Final	82.2	—	—	—	—
Macht results					
Number of wins	4	3	3	1	0
Number of draws	2	1	1	1	1
Number of losses	1	2	1	2	2

Countries are ordered according to their tournament progress. England played a total of seven matches and lost the final against Spain. The team from the Netherlands was eliminated in the semifinal (six matches), Germany was eliminated in the quarterfinals (five matches), Italy was eliminated in the round-of-16 (four matches), and Poland was eliminated in the group stage (hence played only three matches). For events with subscripts, data collection took place on a weekend day (i.e., the comparably lower percentages could be due to: _a_ = data collection on Saturday, _b_ = data collection on Sunday).

**Table 2:** Distribution of Completed Waves Per Participant.

Event	Overall (*N* = 1,012)	England (*n* = 202)	Netherlands (*n* = 204)	Germany (*n* = 204)	Italy (*n* = 201)	Poland (*n* = 201)
Completed 7 waves	98 (9.7%)	**98 (48.5%)**	—	—	—	—
Completed 6 waves	102 (10.1%)	48 (23.8%)	**54 (26.5%)**	—	—	—
Completed 5 waves	113 (11.2%)	16 (7.9%)	36 (17.6%)	**61 (29.9%)**	—	—
Completed 4 waves	191 (18.9%)	12 (5.9%)	26 (12.7%)	54 (26.5%)	**99 (49.3%)**	—
Completed 3 waves	216 (21.3%)	11 (5.4%)	25 (12.3%)	25 (12.3%)	49 (24.4%)	**106 (52.7%)**
Completed 2 waves	120 (11.9%)	4 (2.0%)	20 (9.8%)	24 (11.8%)	24 (11.9%)	48 (23.9%)
Completed 1 wave	112 (11.1%)	4 (2.0%)	30 (14.7%)	26 (12.7%)	18 (9.0%)	34 (16.9%)
Only Baseline	60 (5.9%)	9 (4.5%)	13 (6.4%)	14 (6.9%)	11 (5.5%)	13 (6.5%)

Countries are ordered according to their tournament progress. England played a total of seven matches and lost the final against Spain. The team from the Netherlands was eliminated in the semifinal (six matches), Germany was eliminated in the quarterfinals (five matches), Italy was eliminated in the round-of-16 (four matches), and Poland was eliminated in the group stage (hence participants were only eligible for three waves). Across all countries, 653 participants (64.5%) missed only one wave or less, and 418 participants (41.3%) even participated in all available waves (marked in bold).

Throughout the tournament, we followed a total of *N* = 1,012 participants from (alphabetically): England (*n* = 202), Germany (*n* = 204), Italy (*n* = 201), the Netherlands (*n* = 204), and Poland (*n* = 201). Our sample was 67.1% male, 32.5% female, and 0.4% diverse. Participants’ age ranged from 18 to 74 (*M* = 33.05 years; *SD* = 10.41). More detailed sample characteristics are displayed in Table 3. On average, participants responded to *Md* = 4 event questionnaires (see also Table 2). Following Arend and Schäfer’s simulations for statistical power in multilevel models^17^, detectable effects with *N*_Level1_ = 4 and *N*_Level2_ = 200 were at β = 0.12 (α = 0.05, power = 0.80). Given that the total sample substantially exceeded this number (i.e., sample size on level 1 and 2), multi-level analyses based on this dataset can likely detect even smaller effects.

**Table 3:** Sample Characteristics Across Countries.

Variable	England (*n* = 202)	Germany (*n* = 204)	Italy (*n* = 201)	Netherlands (*n* = 204)	Poland (*n* = 201)
Age	39.02 (12.21)	31.73 (9.59)	34.86 (10.35)	30.54 (8.27)	29.13 (8.08)
Gender (%)					
Male	65.8	69.6	64.2	64.2	71.6
Female	34.2	30.4	35.3	34.3	28.4
Other	0.0	0.0	0.5	1.5	0.0
Sexual orientation (%)					
Heterosexual	89.1	88.7	84.6	85.3	89.6
Homosexual	3.5	4.4	6.0	4.4	4.5
Other	5.0	5.4	9.5	9.3	5.5
Prefer not to say	2.5	1.5	0.0	1.0	0.5
Relationship status (%)					
Single	29.2	45.1	30.8	36.8	34.8
Committed relationship	29.2	29.9	48.3	39.7	42.8
Married	35.6	24.0	19.9	22.5	21.9
Divorced	4.5	1.0	1.0	1.0	0.0
Widowed	1.5	0.0	0.0	0.0	0.5
Education (%)					
Primary education	0.0	0.0	0.0	0.5	0.5
Lower sec. education	2.0	6.4	0.5	2.5	0.0
Upper sec. education	15.3	27.9	37.8	11.3	19.9
Post-sec. non-tert. education	15.8	2.9	0.0	5.9	14.9
Short-cycle tertiary education	2.0	4.9	0.0	3.9	2.5
Bachelor (or equivalent)	49.5	28.9	22.9	44.6	30.8
Master (or equivalent)	13.4	25.0	32.8	29.9	29.4
Doctoral (or equivalent)	2.0	2.9	6.0	1.5	0.5
Other	0.0	1.0	0.0	0.0	1.5
Employment status (%)					
Employed, full-time	61.9	49.5	42.8	56.9	56.7
Employed, part-time	12.9	24.0	16.4	21.6	14.9
Self-employed	6.4	8.3	14.9	6.4	7.0
Unemployed	15.8	16.7	25.4	15.2	21.4
Retired	3.0	1.5	0.5	0.0	0.0
Socioeconomic status^a^	5.31 (1.50)	5.60 (1.53)	5.68 (1.51)	6.25 (1.49)	5.63 (1.46)
Political orientation^b^	4.98 (2.32)	4.94 (2.01)	4.50 (2.11)	5.33 (2.58)	5.98 (2.46)

^a^Socioeconomic status was assessed with the *MacArthur Scale of Subjective Social Status*^51^ asking participants to make a direct comparison between themselves and other people in their country (from 1 = *people who are the worst off* to 10 = *people who are the best off*). ^b^Political orientation was assessed on an 11-point rating scale from 1 = *far left* to 6 = *moderate* to 11 = *far right*.

Please note that the participant samples per country are not entirely representative and differ slightly in average demographic characteristics, particularly with respect to age. To a lesser extent, the samples also differ in socio-economic status and political orientation. Accordingly, cross-country comparisons should be interpreted cautiously, as they may partly reflect sample composition rather than substantive national differences. Researchers interested in cross-country comparisons should thus consider controlling for demographic differences in additional robustness analyses.

### Eligibility and exclusion criteria

All participants in the dataset needed to be at least 18 years old and residents of one of the five participating countries. Note that compared to previous research (see Background), the present dataset goes *beyond* host country residents and also includes spectators from four other *participating* countries. To ensure sufficient exposure to the (results of the) ongoing tournament, only individuals who planned to follow the tournament, either via television, live in the host cities, or via the local media coverage, were eligible for participation. In addition, only individuals that reported to support the country in which they resided were included in the data to prevent cross-country inferences.

As preregistered, we excluded participants who (a) did not pass the Attention Check during the baseline questionnaire (“Please select the third option from the left for this item“; *n* = 25), or (b) stated that they had answered the questions incorrectly in the self-report (*n* = 1). We additionally excluded event questionnaires for which (c) participants gave an incorrect account of the result of the match per measurement wave (i.e., attention check; *k* = 80 across all waves). We did not infer any missing data (e.g., imputation), and we provide datasets in different formats on OSF as well as code to create subsets of these data (e.g., separated by country). The cleaned, final dataset consists of *N* = 1,012 participants and *k* = 3,627 event questionnaires. All subsequent descriptions and values refer to this final dataset.

### Recruitment and payment

Participants were recruited via Prolific, a recruitment platform for online behavior research with higher data quality than other platforms such as MTurk or CloudResearch^18^. Participants received monetary compensation (approximately £7.00 per hour) for the baseline questionnaire and for every completed event questionnaire the day after each match. To maximize participation in as many event questionnaires as possible and to reduce attrition, we incentivized participants financially by issuing bonus payments of £0.20 per event questionnaire for participants that completed at least 80% of all potential event questionnaires. In addition to monetary compensation, participants that responded to all event questionnaires had the chance to win branded tournament merchandise (e.g., match balls, key chains, mascots, etc.) in a lottery after the tournament was finished. Again, this incentive sought to further increase engagement and reduce attrition throughout the data collection period.

### Variable overview

The dataset contains variables from two perspectives: From the event perspective, we assessed (1a) objective event-related variables (e.g., match result, performance ratings) and (1b) subjective match-related variables (e.g., perceived fairness of the match, betting behavior). From the individual perspective, we assessed (2a) trait variables (i.e., individual trait differences, e.g., socio-economic status, Big-5 character traits), and (2b) state variables following match events (e.g., enthusiasm, sexual affect, satisfaction, work motivation). All variables were measured in the national language of the respective sub-sample (i.e., English, German, Italian, Dutch, Polish). If no validated translated scale was available, items were translated and back-translated by bilingual experts, with discrepancies resolved after discussion in the author team. Following recommendations by Gabriel *et al*.^19^, we often used single-item measures to reduce participant burden. We provide a comprehensive list including all variables and the wording of the items in Tables 4–6.

**Table 4:** Variable List for Item-Based or Self-Report Variables.

Variable	Construct	Item
happiness	Happiness	Do you feel happy today?
enthus	Enthusiasm	Today, I’m enthusiastic about my national team.
schadenfreu	Schadenfreude	How much schadenfreude are you experiencing towards England’s last opponent?
envy	Envy	How much envy are you experiencing towards England’s last opponent?
sex_aff	Sexual affect	Since the end of yesterday’s game, how often have you felt sexual desire or felt “turned on”? Since yesterday evening, how often have you felt sexual desire or felt “turned on”?
phys_act	Physical activity	Since the end of yesterday’s game, have you engaged in physical activity (e.g. running, yoga, fitness)? If yes, how intense was this physical activity? Since yesterday evening, have you engaged in physical activity (e.g. running, yoga, fitness)? If yes, how intense was this physical activity?
prosoc_behav_cause	Prosocial behavior (for a good cause)	How willing are you to do something for a good cause today (e.g., donating, volunteering)?
prosoc_behav_help	Prosocial behavior (helping)	How willing are you to help someone today?
use_humor	Use of humor	I found myself joking/laughing a lot today.
match_social	Match-related socializing	Since the end of yesterday’s game, I was keen to talk to other people about this game.
Birg	Basking in reflected glory	Are you wearing, or do you plan to wear, clothing or accessories that display the logo, emblem, or insignia of the English national team today?
sex_beh	Sexual behavior	Since the end of yesterday’s game, how often did you either masturbate or have sex with someone else? Since yesterday evening, how often did you either masturbate or have sex with someone else?
lifesat	Life satisfaction	I am satisfied with my life today.
work_motiv	Work motivation	How motivated are you to work today?
soc_conn	Social connectedness	I feel socially connected with the world around me today.
belief_jw	Belief in a just world	I feel that people get what they deserve.
sex_cogn	Sexual cognition	Since the end of yesterday’s game, how many sexual thoughts and fantasies have you had? Since yesterday evening, how many sexual thoughts and fantasies have you had?
ident_country	Identity with country	How important is being English to you today?
ident_eu	Identity with Europe	I identify as a European today.
ident_team	Identity with the national team	How strongly do you see yourself as a fan of the English national team today?
ident_sport	Identity with football	How strongly do you see yourself as a football fan today?
pol_trust	Political trust	The English government usually does the right thing.
multicult	Support for multiculturalism (reverse coded)	It is better for the country if racial and ethnic groups adapt and blend into the large society.
nat_opt	National optimism	Today, I believe that my country has a positive future ahead.
percept_racism	Perceived racism	I feel like racism is a big issue in my country.
crisis_percept	Perceived crises severity	Today, how severe do you perceive the current world crises (e.g., climate crisis, war in Europe, middle east conflict)?
eval_in	Ingroup evaluation	How do you feel towards English people today?
eval_out	Outgroup evaluation	How do you feel towards foreigners (non-English people) today?
bfi_ext	Extraversion	6 items from BFI-2-S (‘_01’ through ‘_06’)
bfi_agr	Agreeableness	6 items from BFI-2-S (‘_01’ through ‘_06’)
bfi_con	Conscientiousness	6 items from BFI-2-S (‘_01’ through ‘_06’)
bfi_ems	Emotional stability	6 items from BFI-2-S (‘_01’ through ‘_06’)
bfi_ope	Openness	6 items from BFI-2-S (‘_01’ through ‘_06’)
self_esteem	Self-esteem	I have high self-esteem.
free_will	Free will	I believe in free will.
consp_belief	Conspiracy beliefs	Some political and social events are debated (for example 09/11 attacks, the death of Lady Diana, the assassination of John F. Kennedy). It is suggested that the “official version” of these events could be an attempt to hide the truth to the public. Do you think that the official version of the events given by the authorities very often hides the truth?
fanship	General fanship	Do you consider yourself to be a fan of football?
ident_compl	Identity complexity	1. Please name at least 5 and a maximum of 10 social groups or organizations to which you belong (e.g. professions, ethnicity, social background, political parties, sports clubs, religions,…) [random selection of four groups]
		2. Sometimes members of one group also belong to other groups. Please rate this from 0 to 10.
		- Please select 0, if no members of the first group are also members of the second group.
		- Please select 10, if all of the members of the first group are also members of the second group.
		To what extent are people of the group “[GROUP 1]“ also part of the group “[GROUP 2]”? To what extent are people of the group “[GROUP 1]“ also part of the group “[GROUP 3]”? To what extent are people of the group “[GROUP 1]“ also part of the group “[GROUP 4]”?…
outcome_subj	Perceived outcome	Did the result of this game feel like a win or a loss?
referee	Perceived fairness of the game	How fair do you think the refereeing was in this game?
represent	Representativeness of the national team	The national team represents our country and our society.
watching	Watching behavior	Did you watch yesterday’s game? And if yes, how?
alcohol	Alcohol consumption	How much alcohol did you drink during or after yesterday’s game?
betting	Betting behavior	How much money did you bet on yesterday’s game?
age	Age	How old are you?
gender	Gender	Which gender do you identify with?
sex_orient	Sexual orientation	What is your sexual orientation?
ses	Socioeconomic status	Think of this ladder as representing where people stand in England. At the top of the ladder are the people who are the best off, those who have the most money, most education, and best jobs. At the bottom are the people who are the worst off, those who have the least money, least education, worst jobs, or no job. Where would you place yourself on this ladder relative to OTHER people in ENGLAND? Please select the rung that best represents where you think you stand on the ladder.
edu	Educational status	Please select your highest level of education.
relation_status		
relation_duration	Relationship status and duration	My relationship status is ___. For how long have been in this relationship status?
employ_status	Employment status	What is your employment status?
pol_orient	Political orientation	Please indicate your political orientation.
birg_open	Basking in reflected glory (open question)	What was the outcome of this game? Please describe the result in one full sentence.
elig_01	Eligibility check 1	Will you watch at least one game of your national team at the UEFA EURO 2024?
elig_02	Eligibility check 2	Which team do you support for the UEFA EURO 2024?
attent_check	Attention check	Please select the third option from the left for this item.
honest_check	Honesty check	For our research, it is very important to collect reliable data and we are relying on you to have taken part seriously. Please tell us now, if you have taken part seriously (so that we can use your answers for our scientific analysis) or whether you were just clicking through (and we cannot use your answers). Your payment for participating in the study will not be affected by your answer.

As part of the intake questionnaire and all event-related questionnaires, participants responded to a battery of (single-item) questions. This table describes these variables (including variable name in the dataset and scaling). Responses from the intake questionnaire have an ‘_IN’ suffix in the variable name, whereas event-specific questionnaires have the respective timepoint as the suffix (e.g., ‘_t1’ for the first event). A detailed codebook is provided on OSF.

**Table 5:** Variable List for Composite Subjective Variables.

Variable	Description and Coding
sex_scr	Average score across the three variables sexual affect (sex_aff), sexual cognition (sex_cogn), and sexual behavior (sex_beh).
eval_outin_diff	Difference score between the evaluation of outgroup members (eval_out) minus the evaluation of ingroup members (eval_in). Negative values indicate a relative preference for ingroup members (i.e., people of own nationality), whereas positive values indicate a relative preference for outgroup members (i.e., people of other nationalities).
diversity	Average composite score that we captured in a separate study by letting different participants (*N* = 151) from the five countries rate the typicality of the players’ first names, last names, and faces when compared to the average person of the respective nationality on a scale from 1 = *typical* to 7 = *untypical*. For each starting-XI of a respective match day, we computed one joint average score across the three typicality indicators first name, last name, and face. We recoded the resulting scores, so that 1 = *low diversity / high typicality* and 7 = *high diversity / low typicality* for the respective starting-XI. Due to the sensitive nature of this variable, it is not included in the public dataset and will instead be made available to qualified researchers upon reasonable request under appropriate data-use conditions.

We computed composite or difference scores for some of the item-based variables (detailed in Table 4). Responses from the intake questionnaire have an ‘_IN’ suffix in the variable name, whereas event-specific questionnaires have the respective timepoint as the suffix (e.g., ‘_t1’ for the first event).

**Table 6:** Variable List for Objective Event Variables.

Variable	Description and Coding
result	Result of the national team’s match (1 = *win* vs. 0 = *draw* vs. −1 = *loss*)
date	Date when the match was played (format: YYYY-MM-DD)
city	City in which the match was played
stage	1 = *group stage*, 2 = *round of 16*, 3 = *quarterfinal*, 4 = *semifinal*, 5 = *final*
attendance	Number of attendants in the stadium
weekend	Indicator for match day (1 = *weekend match*, 0 = *weekday match*)
weekend_resp	Indicator for data collection day (1 = *weekend*, 0 = *weekday*)
fifa_rank	FIFA world ranking prior to the tournament
fifa_rank_opp	FIFA world ranking prior to the tournament (opponent)
fifa_rank_diff	Difference in FIFA rankings (positive values indicate a higher ranked opponent, negative values indicate a lower ranked opponent)
xg	Expected goals (xG) for the national team are calculated as the aggregated goal scoring probability for each team. The value is calculated for every goal scoring opportunity (i.e., every shot) and considers different factors such as the shot position or the chance quality.
xg_opp	Expected goals (opponent)
xg_diff	Difference in expected goals (positive = higher xG for the national team)
team	National team of interest
team_opp	Opposing national team
goals	Goals
goals_opp	Goals (opponent)
goal_diff	Goal difference of the match (positive = win, 0 = draw, negative = loss)
poss	Ball possession in %
poss_opp	Ball possession in % (opponent)
pass_perc	Pass accuracy in %
pass_perc_opp	Pass accuracy in % (opponent)
pass_att	Pass attempts
pass_att_opp	Pass attempts (opponent)
pass_comp	Complete passes
pass_comp_opp	Complete passes (opponent)
distance	Total distance covered by players
distance_opp	Total distance covered by opponent players
total_att	Total shot attempts
att_target	Shots on target
att_off	Shots off target
att_blocked	Blocked shots
total_att_opp	Total shot attempts (opponent)
att_target_opp	Shots on target (opponent)
att_off_opp	Shots off target (opponent)
att_blocked_opp	Blocked shots (opponent)
woodwork	Shots at goal posts
woodwork_opp	Shots at goal posts (opponent)
corner	Corner kicks
corner_opp	Corner kicks (opponent)
offside	Offside
offside_opp	Offside (opponent)
foul_comm	Fouls committed
foul_comm_opp	Fouls committed (opponent)
ball_rec	Ball recoveries
ball_rec_opp	Ball recoveries (opponent)
tackles	Tackles
tackles_opp	Tackles (opponent)
clear	Clearances
clear_opp	Clearances (opponent)
yellow	Yellow cards
yellow_opp	Yellow cards (opponent)
red	Red cards
red_opp	Red cards (opponent)
tactic	Tactic/formation (e.g., 4-4-2)
tactic_opp	Tactic/formation (e.g., 4-4-2) (opponent)
subs	Substitutions used
subs_opp	Substitutions used (opponent)
player_age	Average age starting XI
player_age_opp	Average age starting XI (opponent)
value	Total market value starting XI in €
value_opp	Total market value starting XI in € (opponent)
penalty	Penalty shootout (0 = *no*, 1 = *yes*)
odds	Decimal odds for the national team to win. Indicates total payout per $1 wagered (including stake). Example: Odds = 2.50 → A $100 bet pays $250 total ($150 profit) if the team wins.
odds_opp	Decimal odds for the opposing national team to win. Indicates total payout per $1 wagered (including stake). Example: 4.00 → A $100 bet pays $400 total ($300 profit) if the opponent wins.
odds_draw	Decimal odds for a draw/tie outcome. Shows total payout per $1 wagered if the match ends in a draw. Example: Odds = 3.20 → A $100 bet pays $320 total ($220 profit) if it’s a draw.
odds_diff	Difference score of odds – odds_opp. Negative values indicate that the respective national team was the favorite for the match, whereas positive values indicate that the opponent team was the match favorite (in terms of betting odds).

For each tournament match, we objectively coded outcome variables. If not otherwise mentioned, team-specific variables refer to the national team of interest. Match data we retrieved from the official webpage of the UEFA (e.g., https://www.uefa.com/uefaeuro/match/2036161–germany-vs-scotland/ for the match between Germany and Scotland on June 14, 2024). Betting data were retrieved from the identical betting provider the day after the matchday. The variable names have the respective timepoint as the suffix (e.g., ‘_t1’ for the first event).

#### Objective match-related variables

The tournament was organized in a preliminary round with 24 teams and a subsequent knockout round with the best 16 teams. For every event (i.e., match), we objectively assessed the following outcome variables. For each tournament match, we coded the *result* of the match with 1 = *win*, 0 = *draw*, −1 = *loss*. In the knockout round, matches could only result in wins (equivalent to progressing to the next round; coded as 1) or losses (equivalent to being eliminated from the tournament; coded as −1). In addition to the match result and match decisiveness, we also assessed more fine-grained performance indicators such as (i) the goal difference (i.e., 0 in case of a draw, positive values in case of a win, negative values in case of a loss), (ii) the expected goals of (and the difference between) the two teams (i.e., aggregated goal scoring probability of each team’s shots in a given match^20,21^), (iii) the commercial decimal betting odds for a win, a draw, and a loss^22,23^, and (iv) the FIFA world ranking of (and the difference between) the two teams (i.e., positive values indicate that participants’ own national team was ranked higher and could be perceived as the favorite for this match). Finally, we coded the (v) cultural diversity of the teams’ starting squads for each event (described in more detail next). In addition, we added a myriad of match-related variables to the dataset after the tournament (e.g., passing accuracy, attendance, yellow and red cards; see Table 6).

#### Team cultural diversity

To assess the cultural diversity of each team, we conducted an additional online study via Prolific in all participating countries (*N*_total_ = 151; *M*_age_ = 31.00 years, *SD* = 10.02; 49.0% male, 49.7% female, 1.3% other). Participants from the main study were not allowed to participate. We asked participants to rate each player (i.e., 26 players in a tournament squad) on how culturally *typical* (a) their first name, (b) their last name, and (c) their face are, compared to the *typical* national first name, last name, and face, respectively (1 = *not at all typical* to 7 = *completely typical*). That is, a highly typical (and non-diverse) name or face would receive higher scores, whereas a non-typical (but highly diverse) name or face would receive lower scores. To capture the player-specific *diversity* score, we reversed these responses and averaged the three indicator scores per participant. Next, to calculate *team diversity*, we calculated the extent to which the starting squad (i.e., the eleven players on the pitch at the start of the match) represented a diverse (i.e., non-typical) team composition for the country they were playing. More concretely, we averaged the diversity scores of the players in the starting squad. Higher values indicated a high(er) cultural diversity (and low typicality) in the starting squad for the respective team and match (see Table 5).

As this measure is construed based on subjective evaluations, we cannot rule out that participants’ typicality ratings might have been influenced by other salient cues (e.g., player familiarity; recent positive performances). The measure should thus be understood as socially perceived national typicality rather than an objective measure of cultural diversity. Hence, we wish to explicitly point out that such measures of typicality^24^ may misrepresent individuals’ actual identity and should rather be interpreted as a result of societal stereotypes. Due to the sensitive nature of this variable, it is not included in the public dataset and will instead be made available to qualified researchers upon reasonable request under appropriate data-use conditions.

#### Subjective match-related variables

We asked the participants after each match how they (i) subjectively perceived the match outcome on a 7-point rating scale (−3 = *felt like a loss* to 0 = *neutral* to + 3 = *felt like a win*). This variable sought to capture participants’ subjective match assessments and to account for certain objective match outcomes (e.g., draws) that felt more like a decisive win (or loss). As expected, wins were subjectively rated much more positively (*M* = 2.09, *SD* = 1.28) than draws (*M* = −0.65, *SD* = 1.73) and losses (*M* = −2.43, *SD* = 1.07), *F*(1, 3,625) = 7,924.20, *p* < 0.001, η_p_^2^ = 0.69—but the remaining variation within losses, wins, and draws illustrates that not all wins (losses) feel equally like a win (loss).

In addition, we asked participants to rate (ii) the perceived refereeing fairness of the match (“How fair do you think the refereeing was in this game?”) on a 7-point rating scale from 1 = *very unfair* to 7 = *very fair* (*M* = 4.96, *SD* = 1.59) and (iii) to what extent the national team represents the respective country (“The national team represents our country and our society”; 1 = *strongly disagree* to 7 = *strongly agree*). To learn more about the behavior and conditions surrounding the match, we also asked participants (iv) where and with whom they had watched the match, (v) whether, and if yes, how much alcohol they had consumed while watching the match (“How much alcohol did you drink during or after yesterday’s game?” from 1 = *very little* to 7 = *very much*), and (vi) whether, and if so, how much money they had bet on the match outcome.

#### Individual Trait Differences

In addition to the demographic variables reported in Table 3, we assessed trait variables on the individual level. We assessed (i) personality traits—openness, conscientiousness, extraversion, agreeableness, and neuroticism—with the 30-item form of the Big Five Inventory–2 (BFI-2-S^25^), (ii) global self-esteem with the single-item self-esteem scale^26^ (“I have high self-esteem”), (iii) free will belief with a single item from 1 = *no belief in free will* to 7 = *absolute belief in free will* (“I believe in free will”^27^), and (iv) conspiracy beliefs with the single-item scale by Lantian *et al*.^28^. As a control, we also assessed (v) general fandom for football prior to the tournament (“Do you consider yourself to be a fan of football?”^29^). Finally, we assessed (vi) identity complexity^30^ (detailed below).

##### Identity complexity

Identity complexity reflects the perceived degree (or the lack) of overlap between social groups in which a person is a member^30^. To assess identity complexity in line with the conceptualization, we asked participants to indicate a minimum of five and a maximum of 10 social groups or organizations to which they feel they belong (e.g., profession, ethnicity, political party, sports club, religion). Next, for every participant, we randomly selected four of their self-reported social groups and asked them to rate the overlap between them sequentially. In six rounds, participants indicated to what extent people of the one group (e.g., green party) are also people of the other group (e.g., fans of the local sport club), rating this overlap from 0 = *none* (i.e., none of the green party members are fans of the local sport club) to 10 = *all* (i.e., all of the green party members are fans of the local sport club). We averaged the six ratings and reversed the values so that higher values indicate less overlap between self-named identity groups, and thus higher identity complexity.

#### Individual state variables

The key components of this longitudinal, event sampling dataset are individual state variables that reflect affective, behavioral, and cognitive day-to-day states and patterns (see Table 4). We used items from validated scales whenever possible and adapted them to our context if necessary (e.g., “…since the end of yesterday’s game” instead of “…this week”).

#### Affective variables

**Fig. 2:**
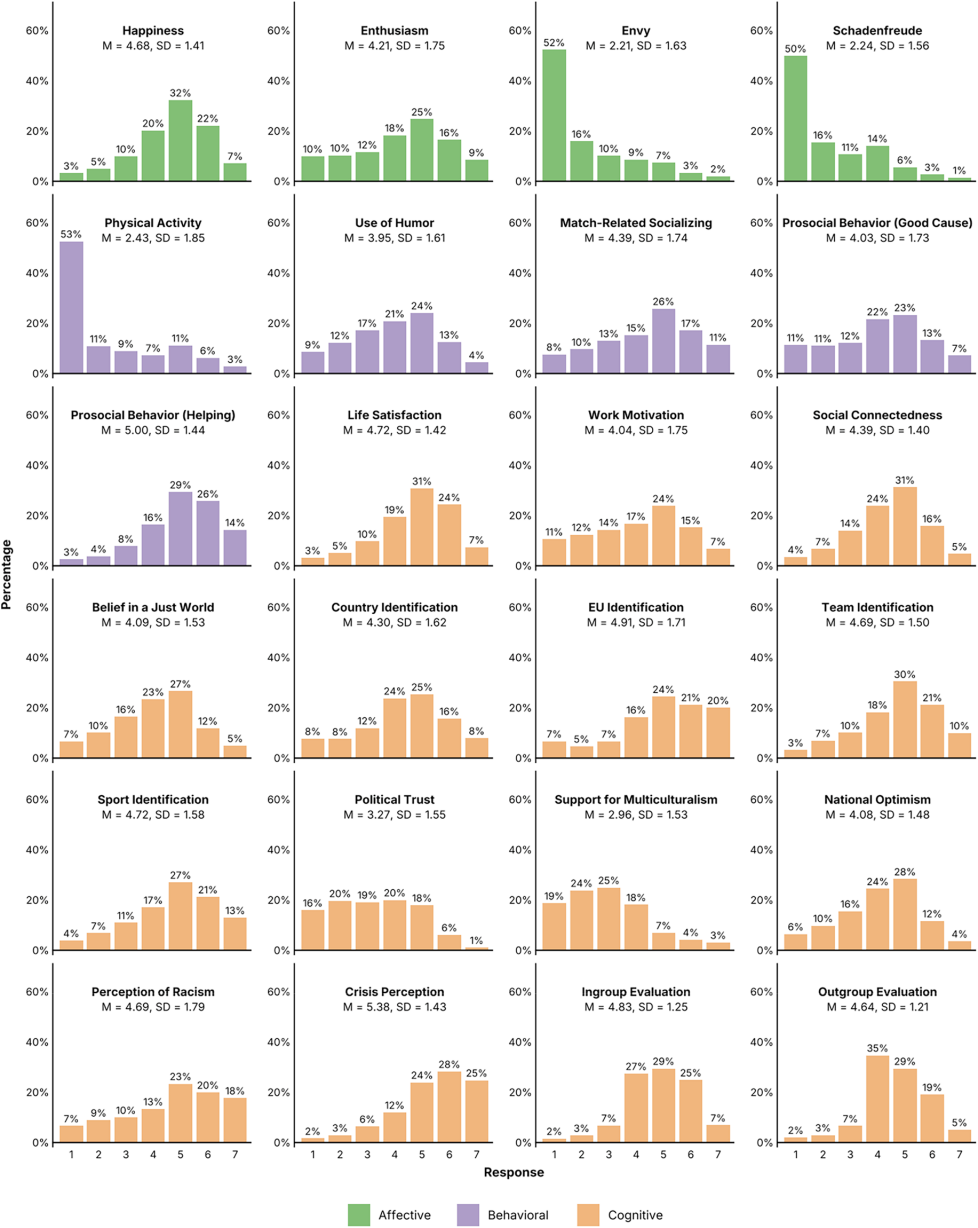
Distributions and Descriptive Statistics for the Continuous Outcome Variables. Sample response distributions for all continuous outcome variables in our dataset. Data is aggregated across all five participating nations and grouped as affective (green), behavioral (purple), and cognitive (orange).

In the affective category (see green plots in Fig. 2), we assessed participants’ (i) happiness^31^ (“Do you feel happy today?”; 1 = *not at all* to 7 = *completely*) and (ii) enthusiasm (“Today, I’m enthusiastic about my national team”; 1 = *strongly disagree* to 7 = *strongly agree*; self-generated). In addition to these positive emotions, we also assessed (iii) schadenfreude^32^ (“How much schadenfreude are you experiencing toward the opponent team?”; 1 = *none at all* to 7 = *a lot*) and (iv) envy toward the opponent team^32^ (“How much envy are you experiencing toward the opponent team?”; 1 = *none at all* to 7 = *a lot*). In addition, we assessed (v) sexual affect^33^ (“Since the end of yesterday’s game, how often have you felt sexual desire or felt ‘turned on’?”; number of occurrences with seven options from 1 = “*0*” to 7 = “*six or more*”).

#### Behavioral variables

In the behavioral category (see purple plots in Fig. 2), we assessed participants’ behavior and behavioral intentions. Participants indicated whether and to what extent they had engaged in (i) physical activity^34^ (“Since the end of yesterday’s game, have you engaged in physical activity (e.g., running, yoga, fitness)? If yes, how intense was this physical activity?”; 1 = *very low intensity* to 7 = *very high intensity*). We also measured participants’ willingness (ii) to do something for a good cause^35^ (“How willing are you to do something for a good cause today [e.g., donating, volunteering]?”) and (iii) to help someone else^35^ (“How willing are you to help someone today?”; 1 = *not at all willing* to 7 = *extremely willing*). We assessed participants’ use of humor (“I found myself joking/laughing a lot today”; 1 = *strongly disagree* to 7 = *strongly agree*; self-generated) and the extent to which they socialized in connection to the match (“Since the end of yesterday’s game, I was keen to talk to other people about this game”; 1 = *not at all willing* to 7 = *extremely willing*; self-generated). We assessed participants’ behavioral affiliation with the national team^36,37^ (“Are you wearing, or do you plan to wear, clothing or accessories that display the logo, emblem, or insignia of the [country] national team today?”; 1 = *not at all* to 7 = *completely*), as well as participants’ sexual behavior^33^ (“Since the end of yesterday’s game, how often did you either masturbate or have sex with someone else?”; number of occurrences with five options from 1 = “*0*” to 5 = “*four or more*”).

#### Cognitive variables

In the cognitive category (see orange plots in Fig. 2), we assessed participants’ (i) life satisfaction^38,39^ (“I am satisfied with my life today”; 1 = *very dissatisfied* to 7 = *very satisfied*), (ii) work motivation (“How motivated are you to work today?”; 1 = *not at all motivated* to 7 = *extremely motivated*; self-generated), (iii) feelings of social connectedness^40^ (“I feel socially connected with the world around me today”; 1 = *strongly disagree* to 7 = *strongly agree*), (iv) belief in a just world^41,42^ (“I feel that people get what they deserve”; 1 = *strongly disagree* to 7 = *strongly agree*), and (v) sexual cognition^33^ (“How many sexual thoughts and fantasies have you had since the end of yesterday’s game?”; number of occurrences with seven options from 1 = “*0*” to 7 = “*six or more*”).

In terms of social identification processes, we assessed participants’ identification (i) with their country^43,44^ (“How important is being [country] to you today?”; 1 = *not important at all* to 7 = *extremely important*), (ii) with Europe^45^ (“I identify as a European today”; 1 = *not important at all* to 7 = *extremely important*); (iii) with the national team^46^ (“How strongly do you see yourself as a fan of the [country] national team today?”; 1 = *not a fan at all* to 7 = *die-hard fan*), and (iv) with football in general^46^ (“How strongly do you see yourself as a football fan today?”; 1 = *not a fan at all* to 7 = *die-hard fan*).

On a socio-political level, we assessed participants’ (i) political trust^47^ (“The [country] government usually does the right thing”), (ii) support for multiculturalism^48^ (“It is better for the country if racial and ethnic groups adapt and blend into the large society”; reverse coded), (iii) national optimism (“Today, I believe that my country has a positive future ahead”; self-generated), and (iv) perceived racism (“I feel like racism is a big issue in my country”; self-generated) on 7-point rating scales from 1 = *strongly disagree* to 7 = *strongly agree*. In a similar way, we assessed participants’ (v) perceived crisis severity (“Today, how severe do you perceive the current world crises [e.g., climate crisis, war in Europe, middle-east conflict]?”; 1 = *not severe at all* to 7 = *extremely severe*; self-generated) and their (vi) ingroup and outgroup evaluation^49,50^ with two items (“How do you feel towards [country] people today?”; “How do you feel towards foreigners (non-[country] people) today?”) on a 7-point rating scale (−3 = *extremely negative*, 0 = *neutral*, + 3 = *extremely positive*).

## Data Record

Our dataset^14^ is publicly shared and available on OSF (10.17605/osf.io/f84g3). The repository provides (1) the primary research datasets (folder name: ‘01_DATA’; including reproducible instructions for creating subsets of the data with selected variables) and supplemental documentation on informed consent forms in all languages and item translations (folder name: ‘02_SUPPLEMENTAL INFORMATION’).

We provide our primary data in the OSF repository (file name: ‘Euro2024_wide.xslx’). A complete list of variable names is provided in Tables 4–6. Our methodology was preregistered before data collection. The preregistration is available here: https://osf.io/a3mrq.

## Technical Validation

To further ensure data quality and validity, we took measures on both the participant level and on the measurement-wave level. First, at the participant level, individuals were excluded entirely if they (a) failed the attention check during the intake questionnaire (“Please select the third option from the left for this item”), or (b) stated in the intake questionnaire that they had answered the questions incorrectly. Second, at the measurement-wave level, individual event questionnaires were excluded if participants (a) indicated that they had answered the questions incorrectly in the event questionnaire or (b) provided an incorrect account of the match result in an open-text field (event-level attention check).

Given the longitudinal panel design, attrition is a potentially important source of bias. We therefore conducted additional analyses to investigate whether non-response is random or systematically related to match outcomes or baseline individual characteristics. For one, we diligently checked for zero-order correlation between all baseline variables and individual participation rate. Reassuringly, the majority of the baseline variables (70.5%) did not correlate with participation rate. In addition, the few significant correlations were of very small size; with the largest baseline correlation (age, *r* = 0.17) explaining less than 3% (*R*^2^ = 0.029) in participation rate across the data collection period of four weeks (see Table 7 for zero-order correlations). We further investigated whether match outcome predicted participation rate (0 = *did not participate*, 1 = *participated*). A mixed-effects logistic regression revealed that participation rate did not differ systematically as a function of the match outcome, *b* = 0.002, *SE* = 0.047, *z* = 0.043, *p = *0.966. Put differently, the estimated probability of participation was virtually identical after losses, draws, and wins, and observed participation rates were 69.9% after wins, 78.3% after draws, and 70.0% after losses.

**Table 7:** Zero-Order Correlations Between Baseline Variables and Individual Participation Rate.

Baseline Variable	*r*	*p*_adj_	Baseline Variable	*r*	*p*_adj_
Age	0.17	<0.001	Physical activity	−0.04	0.326
Extraversion	−0.13	<0.001	Political trust	−0.04	0.337
Conscientiousness	0.13	0.001	Prosocial behavior (helping)	0.03	0.511
Self-esteem	−0.11	0.004	Representativeness of the national team	0.03	0.562
General fanship	0.11	0.006	Support for multiculturalism	−0.03	0.570
Relationship duration	0.10	0.014	Difference in- vs. outgroup	0.03	0.570
Agreeableness	0.09	0.017	Outgroup evaluation	0.03	0.571
Perceived crisis severity	0.09	0.020	Basking in reflected glory	−0.03	0.575
Sexual cognition	−0.09	0.021	Work motivation	0.02	0.629
Sexual score	−0.09	0.025	Identity with the national team	0.02	0.635
Belief in a just world	−0.08	0.029	Free will	0.02	0.732
Sexual behavior	−0.08	0.030	Identity with country	0.02	0.732
Identity with football	0.07	0.101	Life satisfaction	−0.02	0.759
Use of humor	−0.07	0.115	Enthusiasm	−0.02	0.798
Sexual affect	−0.06	0.141	Identity with Europe	0.01	0.834
Socioeconomic status	−0.06	0.152	Political orientation	−0.01	0.845
Identity complexity	−0.06	0.154	Emotional Stability	0.01	0.845
Perceived racism	−0.06	0.154	Happiness	0.01	0.845
National optimism	−0.06	0.169	Prosocial behavior (for a good cause)	0.01	0.845
Conspiracy beliefs	−0.05	0.285	Ingroup evaluation	−0.01	0.882
Social connectedness	0.04	0.326	Openness	−0.00	0.922

*N* = 1,012. Variables are ordered in ascending order of adjusted *p*-values (using FDR method for multiple testing^52^).

Finally, like previously published datasets, we calculated indicators of internal consistency for any scale that contained more than two items. This included Cronbach’s Alpha, McDonald’s Omega, and the proportion of variance explained by a unidimensional factor (see Table 8). We did not conduct additional experiments or analyses to further support or investigate the technical quality of the dataset.

**Table 8:** Summary Statistics for Personality Scales Across All Countries.

Trait	*M*	*SD*	Skew	Kurtosis	ɑ	ω	Var
Extraversion	3.01	0.77	−0.08	−0.18	0.79	0.79	0.49
Agreeableness	3.67	0.65	−0.31	−0.05	0.72	0.73	0.43
Conscientiousness	3.63	0.76	−0.22	−0.49	0.81	0.81	0.51
Emotional Stability	3.24	0.88	−0.13	−0.57	0.85	0.85	0.58
Openness	3.67	0.75	−0.34	−0.41	0.77	0.77	0.46

*N* = 1,012. ɑ = Cronbach’s Alpha, ω = McDonald’s Omega, Var = proportion of variance explained by a unidimensional factor.

## Usage Notes

We recommend using the dataset in ‘long format’ to fully leverage the multilevel structure of the dataset. For more information on how to navigate the OSF repository, read the uploaded README file (file name: ‘260527_readmev1.00.rtf’).

### Declaration of LLM Use

During the preparation of this manuscript, we used ChatGPT 5.1 as a language-editing tool to refine grammar, phrasing, and stylistic clarity of the text that had been written by the authors. All AI-suggested edits were reviewed critically and, where necessary, further revised by the authors, who take full responsibility for the content of the manuscript.

## Data Availability

All anonymized data^14^ is publicly shared on the Open Science Framework page of the research project (10.17605/osf.io/f84g3).

## Code availability

All data, the materials from the study (e.g., surveys, etc.), codebooks, and the code presented in this manuscript are openly available at 10.17605/osf.io/f84g3. We provide a Python script to create filtered subsets of the data on OSF (file name: ‘Euro2024_subsetcreator.ipynb’).
